# Sex Workers Should Be Included in COVID-19 Vaccination Efforts

**DOI:** 10.4269/ajtmh.21-0438

**Published:** 2021-10-25

**Authors:** Gladson Vaghela, Catherine Hermoso, Attaullah Ahmadi, Don Eliseo Lucero-Prisno

**Affiliations:** ^1^ GMERS Medical College, Gandhinagar, Gujarat, India;; ^2^ College of Medicine, Bicol University, Albay, Philippines;; ^3^ Medical Research Center, Kateb University, Kabul, Afghanistan;; ^4^ Department of Public Health, International School of Medicine, Bishkek, Kyrgyzstan;; ^5^ Department of Global Health and Development, London School of Hygiene and Tropical Medicine, London, United Kingdom

## Abstract

As the COVID-19 pandemic takes its toll on citizens across the globe, more people turn to sex work for survival. Because sex work is inherently physical and intimate, sex workers become defenseless against the virus and act as a bridge for transmitting the virus to their clients and society. Often, sex workers are the victims of violence and homelessness, and are devoid of health-care facilities, including HIV treatment, and are frequently exposed to a large number of individuals as dictated by the nature of their work. Their survival instincts would drive them to take part in their usual job to earn money, despite added health risks, to survive and feed their families. Worldwide, sex workers do not fully benefit from the COVID-19 responses, particularly in health, social, and economic aid assistance and services. Hence, it is essential to include this vulnerable population in the COVID-19 vaccination programs to halt the further spread of the virus.

Globally, sex workers belong to a severely marginalized and oppressed population, in which a stigma placed on their work compels them to live outside the law.^1^^–^^3^ While the whole world focuses on the derangements caused by the COVID-19 pandemic, the vulnerable population of sex workers is left behind in the COVID-19 response, especially in the COVID-19 vaccination drive. Sex workers around the world are demanding better access to COVID-19 vaccines as they are further pushed down the COVID-19 vaccine priority list.^4^^,^^5^ This article highlights the need for including sex workers in COVID-19 vaccination efforts across the globe.

The ongoing COVID-19 pandemic has highlighted numerous social and health inequities affecting the sex worker community.^6^^,^^7^ Engaging in work pushes them to the forefront of contracting the virus because it requires physical and intimate contact in its very nature. However, if a sex worker chooses to stop working, surviving on a daily basis is difficult. According to the English Collective of Prostitutes, the majority of sex workers are mothers.^3^ In Chile, more than three quarters of sex workers are the sole breadwinners and typically have at least one dependent.^3^ In India, the COVID-19 outbreak is expected to put 90% of commercial sex workers in red light districts in debt, which they will be unable to repay in their lifetime, according to one study.^8^ Thus, survival instincts drive them to continue working at their current job to earn money and support their dependents, despite the added health risks.

Sex workers are not only at the risk of COVID-19 infection, but they are also susceptible on account of their socioeconomic status and the stigma, discrimination, and abuse they experience in society, as well as a lack of knowledge, services, resources, and support.^6^^,^^7^ The majority of the sex workers work in person without any personal protective equipment during this outbreak; even wearing a mask is not welcomed by their clients.^5^ As a result of a fear of losing clients, female sex workers have been unable to negotiate sex terms and instead have engaged in work without any personal protective equipment to earn money. Such fears have encouraged the concealment of illness and discourage them from seeking treatment. This establishes an “epidemiological bridge,” forming a significant channel for COVID-19 transmission to the general population.^9^

In addition, migrant sex workers, who are either documented or undocumented, are denied housing facilities, with threats of forceful deportation.^7^^,^^10^ Sex workers are unable to access social housing schemes, and many of them are evicted from their homes because they cannot afford rent.^6^ Overcrowding is often common in brothels where sex workers and their children live, making social distancing impossible. Connected to these conditions, infectious disease outbreaks are commonplace in such environments. Often, those who lack adequate housing, who live in poverty, and who lack continued access to health-care services are at greater risk of contracting COVID-19 and becoming affected negatively.^2^^,^^3^^,^^10^ In addition, factors that make female sex workers vulnerable also affect men and transgender sex workers, who must deal with additional issues such as sexual identity and homophobic violence and repression.^6^ Furthermore, sex workers are alienated from social protection schemes and economic relief bills passed by various governments.^6^

Sex workers also share a significant burden of HIV/AIDS. Around the world, 1 in 10 sex workers is estimated to be living with HIV.^11^ Evidence shows that immunocompromised persons, including persons living with HIV/AIDS, might host ongoing severe acute respiratory syndrome coronavirus 2 replication that could enable the development of variants of concern/interest.^12^ This in turn poses direct risks to people’s health and well-being, and has widespread impacts across the population. Sex workers are not only at an increased risk of infection, but also have a high likelihood of the aforementioned consequences of forming an epidemiological bridge by harboring COVID-19. This causes the spilling of the virus among the healthy population and results in an increased economic burden on health-care systems. Excluding sex workers from the COVID-19 vaccination plan not only contradicts public health goals, but also results in long-lasting consequences, prolonging the ongoing pandemic even further.

Hence, the onus is on the government to protect and vaccinate sex workers against COVID-19. This action will prove beneficial for the government and for the general population. Governments could implement the following strategies for vaccinating sex workers: first, include them in the COVID-19 vaccine priority list. The WHO roadmap for COVID-19 vaccine prioritization highlights explicitly the need to include at-risk sociodemographic groups such as the disadvantaged, gender and sexual minorities, people living in extreme poverty, the homeless, low-income migrant workers, vulnerable migrants in irregular situations, nomadic populations, and hard-to-reach populations.^13^ Sex workers are often one among them. The fight for the rights of sex workers intersects with a multitude of other social issues. Studies show that sex workers are more likely to experience homelessness, unemployment, and extreme poverty.^10^

Second, develop effective communication strategies to combat vaccine apprehension and misinformation. Authorities should work with civil societies and nongovernmental organizations that have experience working with sex workers. The organization they trust will help spread the message more effectively than traditional communication channels. Experiences from India demonstrate the importance of civil societies and nongovernmental organizations in debunking myths using WhatsApp groups to raise awareness by posting advisory messages among the sex worker community.^14^ Community leaders ensured that the information reached many hard-to-contact members of the sex worker community through their networks and by word of mouth. Such strategies need to be implemented for an efficient communication channel.

Third, alleviate existing barriers to access health-care services. In health-care settings, sex workers are frequently harassed and denied services, further widening the gap in accessing health-care facilities.^10^ Sensitization of health-care workers will aid in bridging the social divide and will improve sex workers’ overall experience when seeking medical care.^15^ The COVID-19 vaccine with a single dose (rather than a double dose) should be offered, if available, to avoid missing the second dose or the need for follow-up.^16^ This strategy will simplify logistics and reduce the amount of resources needed for a second dose. The digitalization of the COVID-19 vaccination program has led to a digital divide, making registration for vaccination difficult for many.^17^ Hence, onsite registration should be enabled.

In addition, lack of identity documents, including documents with date of birth, proof of residency, and self-identified gender identity, is another barrier to accessing COVID-19 vaccination. Concerns about disclosing their immigration status may discourage migrant sex workers from getting immunized, even when they qualify.^4^ Hence, the use of mobile or pop-up clinics could be incorporated to overcome this barrier. In Vancouver, Canada, pop-up clinics have facilitated civil society and the government to vaccinate sex workers by providing free-of-charge COVID-19 vaccinations.^18^ Sex workers were able to receive their injection at pop-up clinics held at places accessible to them, without producing any proof of identity. Such initiatives will help sex workers avoid any stigma or fear from getting a vaccination.

Access to health-care services is a basic human right. Responding to a health crisis should be inclusive, resulting in equitable outcomes that do not exclude vulnerable groups.^2^^,^^6^ As a global community, we have the capacity to expand vaccination coverage among vulnerable groups and leverage investments in the distribution of COVID-19 vaccines to enhance health infrastructures for everyone, while meaningfully including the sex worker community. As the world faces one of its deadliest zoonotic challenges, how we respond today will decide not just the path of this pandemic, but also who benefits from future pandemics and outbreaks by identifying and resolving existing inequities without abandoning the disenfranchised.
